# Distinct Changes in Metabolic Profile and Sensory Quality with Different Varieties of *Chrysanthemum* (Juhua) Tea Measured by LC-MS-Based Untargeted Metabolomics and Electronic Tongue

**DOI:** 10.3390/foods13071080

**Published:** 2024-04-01

**Authors:** Xing Tian, Haodong Wang, Liang Chen, Hanwen Yuan, Caiyun Peng, Wei Wang

**Affiliations:** 1 TCM and Ethnomedicine Innovation & Development International Laboratory, Innovative Material Medical Research Institute, School of Pharmacy, Hunan University of Chinese Medicine, Changsha 410208, China; acctianxing@hotmail.com (X.T.); whd9974@163.com (H.W.); chenliang_201230@163.com (L.C.); hanwyuan@hnucm.edu.cn (H.Y.); caiyunpeng@hnucm.edu.cn (C.P.); 2 Department of Food and Drug Engineering, School of Pharmacy, Hunan University of Chinese Medicine, Changsha 410208, China; 3 Engineering Technology Research Center of Hunan Province Xiangnan Area Authentic Chinese Medicinal Materials, Yongzhou 425600, China; 4 Confucius Institute, Wonkwang University, 460 Iksandae-ro, Iksan 54538, Republic of Korea

**Keywords:** *chrysanthemum* (Juhua), tea, metabolomics, sensory quality, LC-MS, electronic tongue

## Abstract

*Chrysanthemum* tea, a typical health tea with the same origin as medicine and food, is famous for its unique health benefits and flavor. The taste and sensory quality of *chrysanthemum* (Juhua) tea are mainly determined by secondary metabolites. Therefore, the present research adopted untargeted metabolomics combined with an electronic tongue system to analyze the correlation between the metabolite profiles and taste characteristics of different varieties of *chrysanthemum* tea. The results of sensory evaluation showed that there were significant differences in the sensory qualities of five different varieties of *chrysanthemum* tea, especially bitterness and astringency. The results of principal component analysis (PCA) indicated that there were significant metabolic differences among the five *chrysanthemum* teas. A total of 1775 metabolites were identified by using untargeted metabolomics based on UPLC-Q-TOF/MS analysis. According to the variable importance in projection (VIP) values of the orthogonal projections to latent structures discriminant analysis (OPLS-DA), 143 VIP metabolites were found to be responsible for metabolic changes between Huangju and Jinsi Huangju tea; among them, 13 metabolites were identified as the key metabolites of the differences in sensory quality between them. Kaempferol, luteolin, genistein, and some quinic acid derivatives were correlated with the “astringency” attributes. In contrast, l-(-)-3 phenyllactic acid and L-malic acid were found to be responsible for the “bitterness” and “umami” attributes in *chrysanthemum* tea. Kyoto Encyclopedia of Genes and Genomes pathway enrichment analysis showed that the flavonoid and flavonol biosynthesis pathways had important effects on the sensory quality of *chrysanthemum* tea. These findings provide the theoretical basis for understanding the characteristic metabolites that contribute to the distinctive sensory qualities of *chrysanthemum* tea.

## 1. Introduction

Dietary herbal teas, defined as water-based immersions or decoctions prepared with herbal ingredients, have been used in healthcare and as part of a healthy diet [1]. As a traditional medicine- and food-homologous plant, *Chrysanthemum morifolium* Ramat. (Juhua) has been used for over 3000 years as a herbal tea-based drink, the third-most widely consumed drink after tea and coffee [2]. Notably, drinking *chrysanthemum* tea or beverages was thought to have similar preventive or therapeutic effects on high blood pressure, sore throat, and eye diseases [3]. Modern pharmacological studies have shown that flavonoids, anthocyanins, alkaloids, phenolic acids, and other phytochemicals in *chrysanthemum*, which have antimicrobial, antioxidant, anti-inflammatory, anti-cancer, anti-obesity, nerve-protective, and other functions, provide a theoretical basis for the development of *chrysanthemum* tea and its deeply processed products [4]. However, the literature on *chrysanthemums* mainly focuses on the biological activity, vegetative propagation, and cultivation technology of medicinal *chrysanthemum* [5]. In contrast, there are few reports on edible *chrysanthemum*’s flavor and sensory qualities in dietary herbal teas. 

The taste and sensory qualities of *chrysanthemum* (Juhua) tea are mainly determined by secondary metabolites, such as flavonols, anthocyanins, amino acids, alkaloids, and organic acids [6]. Generally, the variety, region, climate, soil type, and production process of *chrysanthemum* tea determine its sensory qualities, related to its chemical composition [7]. At present, many researchers have systematically analyzed the flavor components of edible *chrysanthemum* using GC-MS, HS-GC-IMS, GC-O, HPLC, sensory evaluation, and other methods [8,9,10]. However, to the best of our knowledge, numerous studies on the sensory qualities and flavor substances of edible chrysanthemum have some problems, such as a single method and lack of multi-omics research [11]. In addition, few studies have been conducted on the core metabolites that influence the sensory qualities of *chrysanthemum* tea.

Metabolomics, as a method of omics, is the science of studying the species, quantity, and changes of metabolites (endogenous metabolites) with molecular weights less than 1500 Da caused by the response of organisms to external stimuli, pathophysiological changes, and gene mutations [12]. Through plant metabolomics, a variety of analysis platforms can be used to study the metabolites of different plant samples after physical or chemical treatment and obtain different meanings, including geographical traceability, food processing, biological activity, etc. [1,13]. In addition, as an intelligent instrument to simulate human taste, electronic tongue systems have been reported to be able to quantitatively and qualitatively analyze the taste of different foods and Chinese herbs [14,15,16]. Thus, the combined analysis of untargeted metabolomics and food flavomics based on LC-MS/MS and an electronic tongue is an excellent method to establish the relationship between *chrysanthemum* tea’s chemical constituents and sensory qualities. However, as far as we know, this multi-omics analysis has not been applied to reveal the key flavor metabolites of different varieties of *chrysanthemum* tea.

The chemical composition, metabolites, and taste of *chrysanthemum* (Juhua) tea may vary somewhat depending on its variety [17]. The objective of this study builds on previous studies [15]. It aims to compare the metabolites and sensory qualities of five different varieties of *chrysanthemum* tea by using LC-MS-based untargeted metabolomics combined with electronic tongue analysis, investigating key differential metabolites associated with sensory quality differences in *chrysanthemum* tea. Importantly, the implementation of this study offers a high-resolution marker for the sensory quality evaluation of *chrysanthemum* tea. This study will provide insight into ascertaining the characteristic metabolites in different varieties of *chrysanthemum* tea.

## 2. Materials and Methods

### 2.1. Chrysanthemum Tea Samples

**Figure 1:**
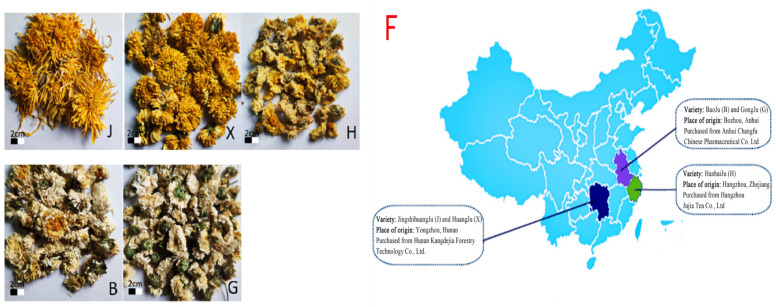
Appearance and origin of five different species of chrysanthemum. Notes: (J) JinshihuangJu; (X) HuangJu; (H) HanbaiJu; (B) BaoJu; (G) GongJu; (F) diagram of the source of five different varieties of *chrysanthemum* tea.

The 5 varieties of *chrysanthemum* tea selected in this study were selected from local producers in Hunan, Anhui, and Hangzhou Provinces, and the 6 biological replicates were found for each variety of *chrysanthemum* tea in the metabolomics study. “JinshihuangJu” (J) and “HuangJu” (X) were selected from local producers in Yongzhou, Hunan Province; “BaoJu” (B) and “GongJu” (G) were collected in the region of Bozhou, Anhui Province; and ”HanbaiJu”(H) was collected from Hangzhou, Zhejiang Province. Detailed information on the *chrysanthemum* tea samples is provided in Figure 1.

### 2.2. Sensory Evaluation for Chrysanthemum Tea Samples

Ten study participants (*n* = 10, 3 males and 7 females, 18–21 years of age) were recruited from the Hunan University of Chinese Medicine’s internal student panel without any exclusion parameters aside from being in good health, nonsmoking, and not currently taking medication. In order to eliminate the influence of environmental factors on the sensory evaluation results, sensory testing experiments were conducted in a single room at 20–25 °C and were performed in the morning (9:00 to 11:00 a.m.). According to the National Standards of China (GB/T23776-2018) [18], all subjects were trained 4 times in 2 weeks, and the sensory attributes used for the sensory quality evaluation of *chrysanthemum* tea samples were screened and identified. Briefly, the different varieties of *chrysanthemum* tea were weighed (2.00 ± 0.05 g), brewed with 150 mL of boiling water for 30 min, and then filtered out with gauze to prepare samples to be immediately evaluated by the panel. Each *chrysanthemum* tea sample was triplicated and randomly coded at room temperature, and each sensory participant tasted a total of 15 tea samples. Each sensory participant scored the *chrysanthemum* tea samples for aroma, astringent sensation, and three taste characteristics (bitterness, umami, and sweetness). The intensity of the sensory attributes was scored on a six-point scale from “0” (unobservable) to “5” (the strongest observable) [19,20]. Still pure water was used as a neutralizer between the *chrysanthemum* tea samples. All participants signed a written informed consent form to participate in this study before the evaluation, and the sensory panels did not communicate with each other during the whole sensory evaluation. 

### 2.3. Electronic Tongue Analysis for Chrysanthemum Tea Samples 

The taste attributes of the five different varieties of *chrysanthemum* tea were determined using a TS-sa402b electronic tongue system (Intelligent Sensor Technology Co., Ltd., Atsugi, Japan) with wide-area selection-specific artificial lipid membrane sensors. The changes in the membrane potential caused by electrostatic interactions or hydrophobic interactions between various flavor substances and artificial lipid membranes were detected to evaluate the eight sensory attributes (saltiness, bitterness, sourness, umami, umami aftertaste, astringency, bitter aftertaste, and astringency aftertaste) of each sample. The taste sensory index data in the samples to be tested were calculated according to the absolute value of the lipid membrane potential of each artificial sensor on the basis of the potential of the solution, in which the output value of the reference solution is called the tasteless point. In this study, as shown by the tasteless blank sample, the tasteless points for different taste components were 0, and values higher than these tasteless points were considered to be meaningful. Specifically, the (2.00 ± 0.05 g) *chrysanthemum* tea sample was accurately weighed and brewed with 150 mL of boiling water for 30 min. Then, the tea broth was filtered through 3 layers of gauze to obtain the sample to be cooled to room temperature before being detected by the TS-sa402b electronic tongue system. In this study, each sample was cycled 4 times, and the mean values of the last three cycles were used for the statistical analysis and analyzed using TS-sa402b library search software (Intelligent Sensor Technology Co., Ltd., Atsugi, Japan). Data collected by the electronic tongue were calculated according to the formula as described. In order to ensure that the sensors were working in the correct mV range, each sensor was checked before measurement, according to the manufacturer’s instructions. 

### 2.4. HPLC Analysis for Chrysanthemum Tea Samples

All *chrysanthemum* tea samples were ground separately into 100-mesh-size fine powders. A 25 ± 0.01 mg sample of each *chrysanthemum* tea was extracted using ultrasonic extraction (power: 300 W, frequency: 45 kHz) (SB-5200DTD; Scientz, Ningbo, China) with 25 mL of ultrapure water at room temperature for 40 min. The available range for extraction temperature was from 0 to 80 °C. After ultrasonic extraction, the sample was centrifuged at 3000 rpm for 10 min to collect the supernatant. Subsequently, the sample was shaken and filtered to obtain the filtrate through a 0.22 μm microfiltration membrane before HPLC analysis.

The contents of chlorogenic acid, luteolin, and 3,5-O-dicaffeyl quinin acid in the *chrysanthemum* tea samples were analyzed using a high-performance liquid chromatography (HPLC) analytical method, following the *Pharmacopoeia of the People’s Republic of China*. The HPLC system had pumps and an autosampler (Agilent 1260 Infinity II Prime liquid chromatography system, Agilent Technologies, Inc., Palo Alto, CA, USA). An HPLC column (250 × 4.6 mm, 5 μm particle size, Welch Technologies, Shanghai, China) was used. An auto-injector injected 10 μL of the test solution into the HPLC system, and the flow rate was 1.0 mL/min. The mobile phase consisted of mobile phase A [H_2_O containing 0.05% (*v*/*v*) phosphoric acid] and mobile phase B [0.1% (*v*/*v*) acetonitrile]. Additionally, the gradient elution was as follows: 0–11 min, the gradient of phase B increased from 10% to 18%, 11–30 min, the gradient of phase B increased to 20%, 30–40 min, the gradient of phase B was maintained at 20% for 10 min, 40–45 min, the gradient of phase B continued to rise to 95%, 45–60 min, and the gradient of phase B was maintained at 90% for 15 min. Furthermore, samples (10 μL) were eluted at 0.8–1.0 mL/min, and the column oven was kept at 30 °C. The HPLC chromatograms of the five different varieties of *chrysanthemum* tea can be found in the Supplementary Materials (Figure S1).

### 2.5. Measurement of Total Polyphenols and Total Flavonoids in Chrysanthemum Tea 

To determine total polyphenols and total flavonoids, 3.0 ± 0.01 g of each *chrysanthemum* tea powder sample was mixed with 120 mL of distilled water in a 250 mL round-bottomed flask and extracted at 90 °C for 40 min in a water bath. After extraction, each extracted solution was centrifuged at 5000 r/min for 5 min, concentrated at 65 °C under reduced pressure for 1 h, and then freeze-dried. After drying, the extracted powder was stored immediately at −20 °C for future analysis. The total polyphenol contents of the *chrysanthemum* tea samples were measured according to the Folin-Denis method. Briefly, 0.01 g of each extracted *chrysanthemum* tea powder was mixed with 1.0 mL of Folin-Ciocâlteu reagent (1.0 mol/L) and reacted at room temperature for 1 min. Then, 8 mL of 10% sodium carbonate (100 g/L) was mixed well, and the mixture was left in a dark place for 2 h. The absorbance was measured at 760 nm using a spectrophotometer (UV-1700, Shimadzu, Kyoto, Japan). The results were expressed as milligrams of gallic acid equivalents per gram of dry matter (mg GAE/g), using gallic acid as a reference standard. The total flavonoid contents of the *chrysanthemum* tea samples were determined according to the aluminum chloride complex formation method and expressed as milligrams of rutin equivalents per gram of extracted *chrysanthemum* tea powder in dry weight (mg RE/g).

### 2.6. LC-MS/MS-Based Untargeted Metabolomics Analysis

The *chrysanthemum* tea samples (80 ± 0.1 mg) were immediately frozen in liquid nitrogen and ground into fine powder with a mortar and pestle. Then, 1000 μL of methanol/acetonitrile/H_2_O (2:2:1, *v*/*v*/*v*) was added to the homogenized solution for metabolite extraction. The mixture was centrifuged for 20 min (14,000× *g*, 4 °C). The supernatant was dried in a vacuum centrifuge. For LC-MS analysis, the samples were re-dissolved in 100 μL of acetonitrile/water (1:1, *v*/*v*) as a solvent and centrifuged at 14,000× *g* and 4 °C for 15 min, after which the supernatant was injected. The untargeted metabolomics analysis of the *chrysanthemum* tea samples was performed using a UHPLC system (1290 Infinity LC, Agilent Technologies, Santa Clara, CA, USA) equipped with a binary pump and a C18 column (2.1 mm × 100 mm, i.d., 1.8 μm, Agilent) operated at 40 °C. Mobile phase A consisted of 25 mmol L ammonium acetate and 0.5% formic acid in water, and mobile phase B was methanol. Additionally, the gradient elution was as follows: 0–0.5 min, 5% B; then B changed to 100% linearly from 0.5 to 10 min; 10–12.0 min, B was maintained at 100%; from 12.0 to 12.1 min, B changed linearly from 100% to 5%; 12.1–16 min, B was maintained at 5%. The sample was placed in an automatic sampler at 4 °C during the analysis. The separated components were then detected with a quadrupole time-of-flight device (AB Sciex TripleTOF 6600, Shanghai Applied Protein Technology Co., Ltd., Shanghai, China). To avoid the effects of instrument fluctuations, a random sequence was used to analyze the samples. QC samples were inserted into the sample queue to monitor and evaluate the stability and reliability of the data.

The ESI source parameters were set as follows: ion source gas 1 (Gas1) as 60, ion source gas 2 (Gas2) as 60, curtain gas (CUR) as 30, source temperature 600 °C, ion spray voltage floating (ISVF) ± 5500 V. In MS-only acquisition, the instrument was set to acquire over the *m*/*z* range 60–1000 Da, and the accumulation time for the TOF MS scan was set to 0.20 s/spectrum. In auto MS/MS acquisition, the instrument was set to acquire over the *m*/*z* range 25–1000 Da, and the accumulation time for the product ion scan was set to 0.05 s/spectrum. Moreover, the product ion scan was acquired using information-dependent acquisition (IDA), with high-sensitivity mode selected. The parameters were set as follows: the collision energy (CE) was fixed at 35 V with ±15 eV; the declustering potential (DP) was 60 V (+) and −60 V (−); we excluded isotopes within 4 Da; 10 candidate ions were monitored per cycle.

### 2.7. Metabolomics Data Acquisition and Analysis

The TIC diagram in the ESI-positive and -negative modes for the five different varieties of *chrysanthemum* tea is shown in Figure S2. The raw MS data were converted to MzXML files using ProteoWizard MSConvert before importing them into the freely available XCMS ^plus^ software (https://sciex.com/cl/products/software/xcms-plus-software, accessed on 12 March 2024) (Sciex, Framingham, MA, USA). For peak picking, the following parameters were used: centWave *m*/*z* = 10 ppm, peak width = c (10, 60), prefilter = c (10, 100). For peak grouping, bw = 5, mzwid = 0.025, minfrac = 0.5 were used. The R-package CAMERA (Collection of Algorithms for MEtabolite pRofile Annotation) was used for annotating isotopes and adducts. In the extracted ion features, only the variables with more than 50% of the nonzero measurement values in at least one group were kept. Compound identification of metabolites was performed by comparing the accuracy of *m*/*z* values (<10 ppm) and MS/MS spectra with an in-house database established with available authentic standards.

After normalizing to total peak intensity, the processed data were analyzed using an R package (ropls), where they were subjected to multivariate data analysis, including Pareto-scaled principal component analysis (PCA) and orthogonal partial least squares discriminant analysis (OPLS-DA). The 7-fold cross-validation and response permutation testing were used to evaluate the robustness of the model. Furthermore, the variable importance in projection (VIP) value of each variable in the OPLS-DA model was calculated to indicate its contribution to the classification. In this study, metabolites with VIP values > 1.0 were further subjected to Student’s *t*-test (*p*-value < 0.05) at the univariate level to measure the significance of each metabolite.

### 2.8. Bioinformatics Analysis

The statistically significant differences in metabolites among the varieties of *chrysanthemum* tea (VIP values > 1 in the OPLS-DA model and *p* < 0.05) were screened for bioinformatics analysis, including hierarchical clustering analysis, correlation analysis and pathway analysis. The hierarchical clustering analysis was also carried out using TBtools software V2.2030 (TBtools, Guangzhou, China). Moreover, the differentially expressed metabolites were matched against the Kyoto Encyclopedia of Genes and Genomes (KEGG) database by the KEGG Automatic Annotation Server (KAAS; website: https://www.genome.jp/tools/kaas/, accessed on 30 July 2023). Values of *p* < 0.05 in Fisher’s exact test were considered statistically significant.

### 2.9. Statistical Analysis

Each experiment was conducted in triplicate, and the results are presented as the mean ± standard deviation. The multiple comparisons of the five varieties of *chrysanthemum* tea groups were calculated by one-way analysis of variance (ANOVA) with Duncan’s test for Statistics 25.0 software (IBM, Chicago, IL, USA). The hierarchical clustering analysis was carried out using TBtools software (TBtools, Guangzhou, China). SIMCA-P 14.1 multivariate statistical software (Umetrics, Umea, Sweden) was specifically used for radar plot generation, PCA, OPLS-DA, and other graphical presentations.

## 3. Results and Discussion

### 3.1. Sensory Quality of the Five Different Varieties of Chrysanthemum Tea

The differences in sensory attributes of the five different varieties of *chrysanthemum* tea were detected by the electronic tongue system. As shown in Table 1, the astringency, bitterness, and umami of the five different varieties of *chrysanthemum* tea were significantly higher than the tasteless point (*p* < 0.05). Therefore, the astringency, bitterness, and umami indices could be used as effective sensory indices for the five *chrysanthemum* tea varieties. As a unique-scented tea health drink, *chrysanthemum* tea has gradually entered the field of view of more consumers. However, *chrysanthemum* tea has astringency, which cannot provide some consumers with oral pleasure, resulting in the low market recognition of simple *chrysanthemum* tea products [15]. It is worth noting that astringent sensation is an important sensory perception of *chrysanthemum* tea, with hydrolyzed and concentrated tannins responsible for this property [21]. Moreover, astringent sensation, one of the most complex oral sensations, is an important essential affecting the sensory quality of food, tea, and other beverages [22]. The astringency index of the HuangJu sample (X) was significantly higher than that of the other three species except for the BaoJu sample (B) (*p* < 0.05). Meanwhile, the umami aftertaste of the HuangJu sample (X) was also significantly higher than that of the other four varieties of *chrysanthemum* tea (*p* < 0.05). In fact, astringent sensation is not a basic taste but, rather, a feeling of convergence caused by the coagulation of proteins in the oral mucosa, which is the result of stimulating the oral nerve endings [22]. Notably, this dry, wrinkled taste occurs when drinking tea or consuming g other foods containing polyphenols. 

In addition, the three taste qualities (including bitterness, umami, and sweetness), astringency, and aroma intensity of the five different varieties of *chrysanthemum* tea were quantified using a sensory evaluation panel consisting of ten trained individuals (Table 2). The results showed no significant differences in umami taste among the five different varieties of *chrysanthemum* tea (*p* < 0.05), while the astringency, bitterness, and aroma of “HuangJu” (X) were significantly (*p* < 0.05) higher than those of the other four species of *chrysanthemum*. Additionally, the results of sensory evaluation also showed that astringency and bitterness could be used as effective sensory attributes of *chrysanthemum* tea. The astringent sensation is mainly caused by the precipitation or aggregation of polyphenols associated with proteins in saliva. In fact, chlorogenic acid and other phenolic acids are easily soluble in *chrysanthemum* tea brewing, thus enhancing acidity and influencing the tasting process of other polyphenols [16]. Therefore, further analysis of the main active compounds and untargeted metabolomics analysis of the five different varieties of *chrysanthemum* tea were carried out.

### 3.2. Comparison of the Contents of Main Active Compounds

Flavonoids are phenolic compounds that are widely present in *chrysanthemum* tea. As shown in Table 3, among the five different varieties of *chrysanthemum* tea, both total polyphenols and total flavonoids had the highest concentrations in HuangJu (X), which were 58.91 ± 0.02 mg GAE/g dw and 201.07 ± 0.05 mg RE/g dw, respectively, significantly higher than in the other four kinds of *chrysanthemum* tea (*p* < 0.05). In contrast, the lowest content of phenolic acids was found in BaoJu (B), which was significantly lower than that of the other four *chrysanthemum* teas (*p* < 0.05). According to Table 2 and Table 3, the results also confirmed that the contents of flavonoids and polyphenols were positively correlated with bitterness and astringent sensation. In fact, chlorogenic acid, luteolin, isochlorogenic acid, and other phenolic acids are the major components of *chrysanthemum* tea [23]. Phenolic acids are responsible for *chrysanthemum* tea’s distinctive color and taste, and the bioactive components contribute to its antibacterial, antiviral, antioxidant, antihypertension, and hypolipidemic activities [24]. Notably, the astringent sensation produced by drinking *chrysanthemum* tea is caused by the polyphenol–protein complex reaction [25]. HuangJu (X) had the highest contents of three phenolic acids and astringent sensation, indicating that the major bioactive substances in the five varieties of *chrysanthemum* tea showed highly comparable curves with the sensory quality data. In fact, due to these differences in variety and origin, HuangJu (X) may be quite different from other types of *chrysanthemum* tea in terms of chemical compounds and sensory characteristics. Hence, follow-up untargeted metabolomics analysis was conducted to provide in-depth information regarding the relationships between the characteristic metabolites and sensory qualities of *chrysanthemum* tea by identifying metabolites in five different varieties.

### 3.3. Untargeted Metabolomics Analysis

**Figure 2:**
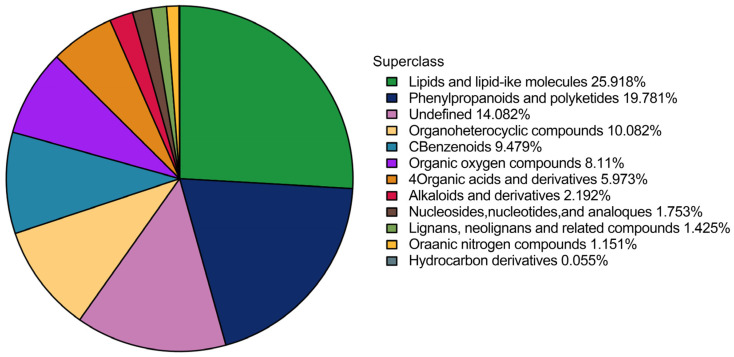
The proportions of the various metabolites in *chrysanthemum* tea samples. Notes: Different color blocks represent items belonging to different chemical classifications, and the percentage represents the items belonging to each chemical classification. The number of metabolites is given as a percentage of all identified metabolites. Metabolites that have no chemical classification are defined as undefined.

Untargeted metabolomics combined with multivariate analysis was applied to investigate the differences in metabolites between the five varieties of *chrysanthemum* tea, and to identify critical metabolites responsible for metabolomic variations caused by different varieties of *chrysanthemum* tea. This study identified metabolites in *chrysanthemum* tea samples according to the in-house database (Shanghai Applied Protein Technology, Shanghai, China) [26]. After pre-treatment and data normalization, 1105 and 670 metabolites were identified from the total ion chromatogram of UPLC-QTOF-MS in positive and negative ion modes, respectively. According to their chemical taxonomy, all metabolites (identified by combining positive and negative ions) were classified and performed on the attribution information. The proportions of the various metabolites are shown in Figure 2, including 473 lipids and lipid-like molecules (25.918%), 361 phenylpropanoids and polyketides (19.781%), 184 organoheterocyclic compounds (10.082%), 173 benzenoids (9.479%), 148 organic oxygen compounds (8.11%), 109 organic acids and derivatives (5.973%), 40 alkaloids and derivatives (2.192%), 32 nucleosides and analogs (1.753%), 26 lignans, neolignans, and related compounds (1.425%), 21 organic nitrogen compounds (1.151%), 1 hydrocarbon derivative (0.055%), and 257 other undefined compounds (14.082%).

**Figure 3:**
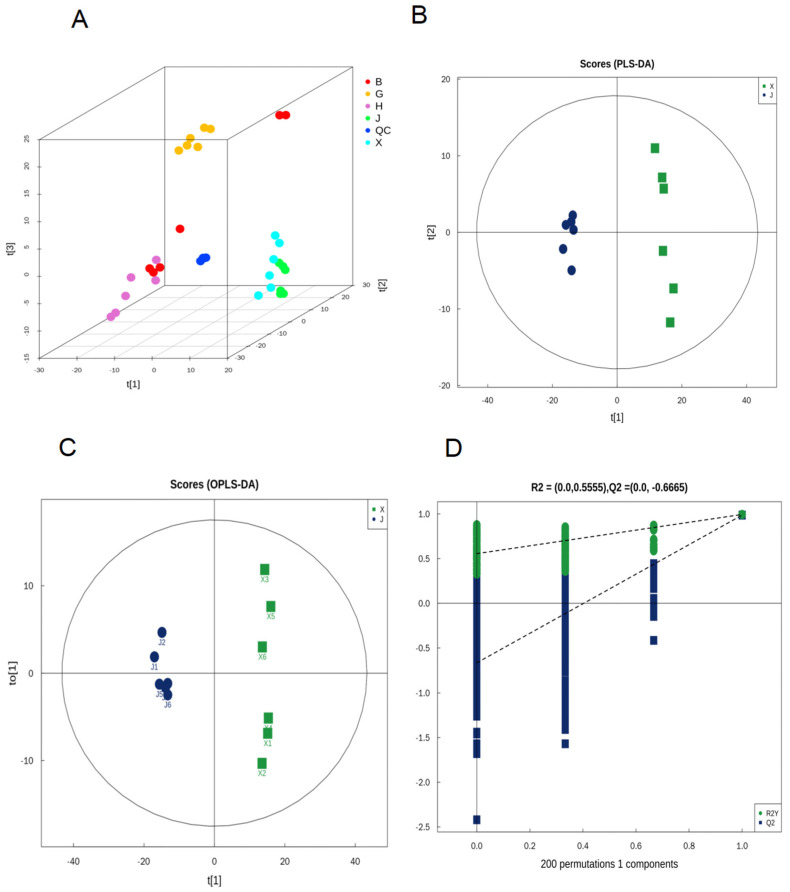
Multivariate analysis of *chrysanthemum* tea samples: (**A**) The 3D PCA of five different species of chrysanthemum. (**B**) The PLS-DA plot (X vs. J), R2X = 0.751, R2Y = 0.994, Q2 = 0.979. (**C**) The OPLS-DA score plot (X vs. J), R2X = 0.753, R2Y = 0.994, Q2 = 0.987. (**D**) Permutation plot of OPLS-DA, R2 = (0.0, 0.5555), Q2 = (0.0, − 0.6665). Notes: (J) JinshihuangJu; (X) HuangJu; (H) HanbaiJu; (B) BaoJu; (G) GongJu.

The principal component analysis (PCA), partial least squares discriminant analysis (PLS-DA), and orthogonal projections to latent structures discriminant analysis (OPLS-DA) methods have been used to identify combinations of metabolites accounting for the most variance, and to visualize sample cluster trends in tea [27]. All metabolites were subjected to multivariate analysis using SIMCA-P 14.1 multivariate statistical software. As an unsupervised data analysis method, the PCA can reflect variability between and within sample groups. As shown in Figure 3A, when using all of the data on the metabolite ion features of five *chrysanthemum* tea samples, the QCs were clustered together on the PCA score plots, which revealed that the data variability was small. It is noteworthy that the X (“HuangJu”) and J (“JinshihuangJu”) *chrysanthemum* tea samples were similar in the PCA but were separated in PLS-DA (Figure 3A,B). In addition, these two kinds of *chrysanthemum* tea samples are from the same origin in Yongzhou, Hunan Province. Therefore, to obtain a higher level of population separation and better understand the differences between different varieties of *chrysanthemum* tea, OPLS-DA was used for classification and to confirm the separation between the “JinshihuangJu” (J) and “HuangJu” (X) tea samples in terms of the various significant parameters. Based on OPLS-DA, the separation trends between J and X samples showed more obvious variations (Figure 3C), and the cross-validation with 200 permutation tests indicated that this OPLS-DA model was reliable, with the intercepts of R^2^ and Q^2^ being 0.5555 and −0.6665, respectively (Figure 3D). Differential metabolites of J and X samples were found by OPLS-DA, and variable importance in projection (VIP > 1, *p* < 0.01) and |log2 (fold change)| values > 1.5 were used for screening. In both positive and negative ion modes, 143 VIP metabolites were responsible for metabolic changes between the J and X samples, including 13 metabolites, 40 flavonoids and flavone glycosides, 31 acids, 22 ketones, 8 esters, 7 amino acids, 7 glycosides, 6 alkaloids, 6 alcohols, and 16 other metabolites. (Table S1).

**Figure 4:**
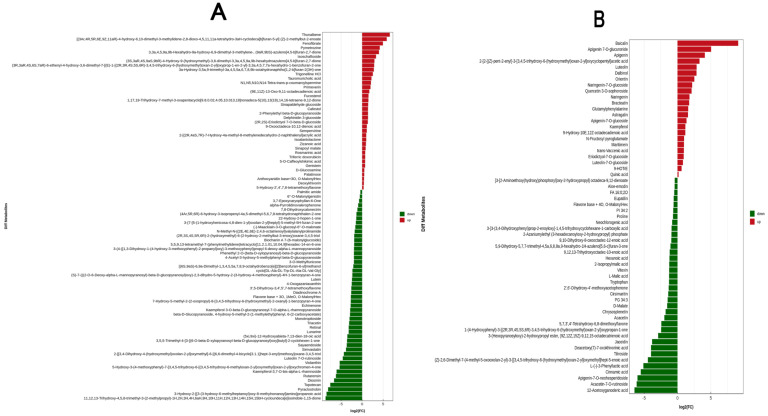
Multiple analysis of significant differences in metabolite expression between X and J samples (VIP > 1, *p* < 0.01): (**A**) In positive ion mode. (**B**) In negative ion mode. Notes: the x-coordinate represents the log2 FC value of the differential metabolite, that is, the logarithmic value of the differential multiple of the differential metabolite is taken as the base 2, and the ordinate axis represents significant differential metabolites. The red indicates upregulated differential metabolites, while green indicates downregulated differential metabolites.

A multiple analysis was applied to visualize the differences in these critical metabolites between the “JingshihuangJu” (J) and “HuangJu” (X) samples (Figure 4). The x-coordinate represents the log2 FC value of the differential metabolite, and each row represents a critical metabolite. The red and green bar charts correspond to differential metabolites up and down, visually showing the changes in the multiple metabolic differences identified as significant.

Polyphenols are phytonutrients, the most abundant contents in *chrysanthemum* tea, containing flavonoids, phenolic acids, lignans, and stilbenes [3]. Isochlorogenic acid C, luteolin, apigenin-7-glucoside, chlorogenic acid, apigenin, and cryptochlorogenic acid play important roles in distinguishing different chrysanthemum varieties [11]. According to Figure 4, the most abundant marker metabolites in J and X samples were flavonoids and flavone glycosides. For example, the abundance of thunalbene, isoschaftoside, delphinidin 3-glucoside, primeverin, genistein, astragalin, bracteatin, maritimein, apigenin, kaempferol, luteolin, naringenin-7-O-glucoside, apigenin-7-O-glucoside, apigenin-7-O-glucuronide, naringenin, orientin, luteolin-7-O-glucoside, baicalinEriodictyol-7-O-glucoside, and quercetin 3-O-sophoroside was upregulated, while the contents of kaempferol-3,7-O-bis-alpha-L-rhamnoside, rutarensin, violanthin, luteolin 7-O-rutinoside,3′,5-dneohesperidoside, chrysosplenetin, acacetin-7-O-rutinoside, jaceidin, 5,7,3′,4′-tetrahydroxy-6,8-dimethoxyflavone, vitexin, cirsimaritin, and eupatilin were downregulated. In fact, flavonoids and flavonoid glycosides play a central role in all aspects of plant life, particularly in the interactions between the plant and the environment, and determine taste and biological activity [13]. The flavonoid glycoside is an important astringency compound in *chrysanthemum* teas, with a velvety taste and an oral coating sensation [16]. In this study, the data of untargeted metabolomics analysis showed a correlation with the taste index of the electronic tongue analysis, where the luteolin and apigenin had the highest contributions to the differences between these *chrysanthemum* teas, as indicated by high VIP values, which were responsible for tea infusion’s bitterness and astringency [28]. Moreover, the abundance of luteolin and apigenin in X (“HuangJu”) samples was significantly different that in J (“JinshihuangJu”) samples (Figure 4, *p* < 0.01). Thus, the degradation of flavonols and flavonoids in different *chrysanthemum* varieties may play a crucial role in forming *chrysanthemum* tea’s flavor. In practice, *chrysanthemum* tea contains extraordinarily high levels of flavonoids that contribute to the tea’s health benefits and flavor characteristics [29]. In fact, phenolic compounds, such as flavonoids and isoflavonoids, are the focus of health research. However, many flavonoids and xenoflavones have bitterness and astringency, which are undesirable and unavoidable to consumers, hindering their use as bioactive substances in foods and beverages [30]. Understanding edible *chrysanthemum*’s “bitterness and astringency motif” might prevent the introduction of bitter taste and astringent sensation in the design of functional foods enriched in bioactive (iso)flavonoids. Therefore, improving the bioavailability and reducing the bitterness and astringency of *chrysanthemum* tea by modifying its flavonoids without affecting its sensory quality will be one of the directions of in-depth, comprehensive research in the future.

### 3.4. Identifying the Core Metabolites

**Figure 5:**
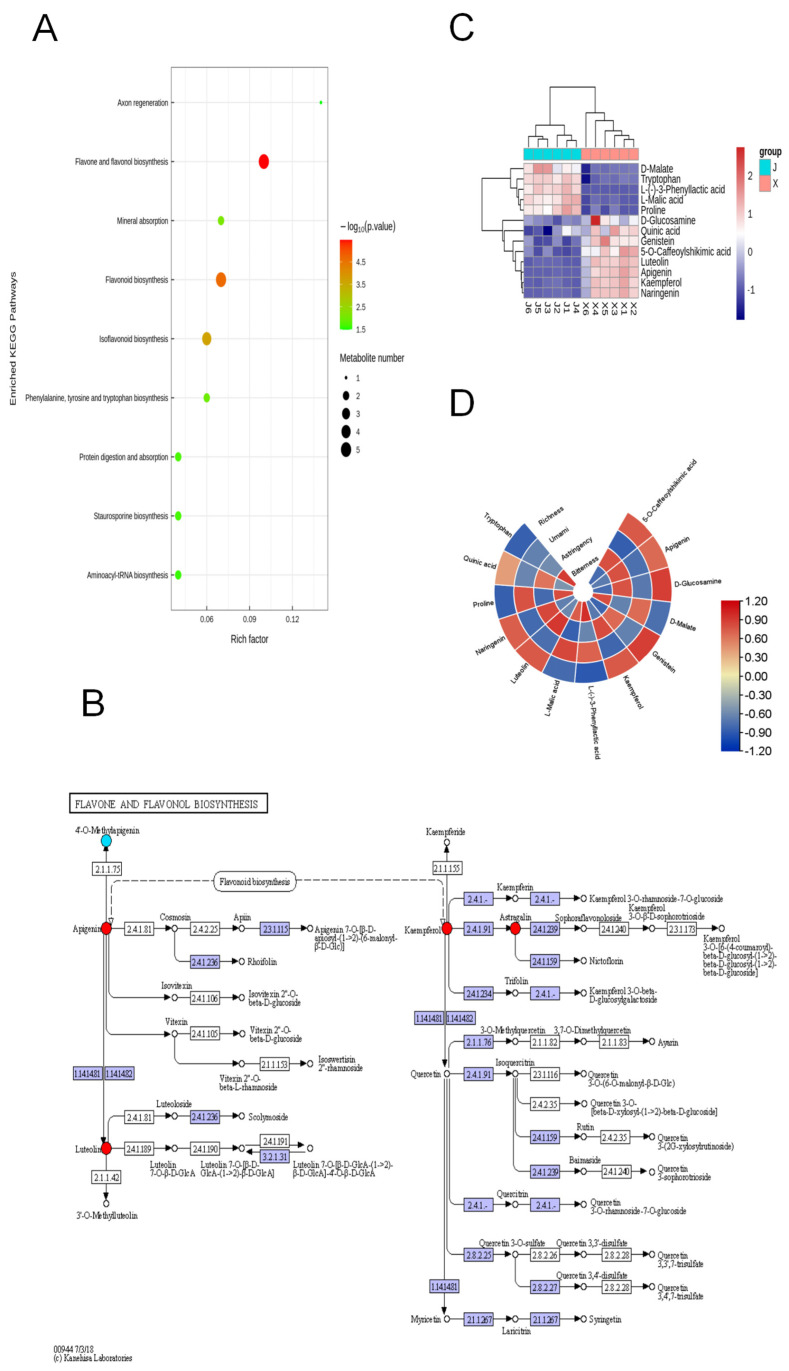
Identifying the core metabolites: (**A**) The KEGG enrichment pathway bubble map between X and J samples. (**B**) The flavone and flavonol biosynthesis pathways. (**C**) Heatmap analysis of critical metabolites in the flavone and flavonol biosynthesis pathways. (**D**) Associations between taste characteristics and metabolite data. Note: (**A**) Each bubble in the figure represents a metabolic pathway (the top 20 with the highest significance were selected according to *p*-value). The horizontal coordinate where the bubble is located and the bubble size represent the influence factor size of the path in the topology analysis, and the larger the size, the larger the influence factor. The vertical coordinate where the bubble is located and the bubble color represent the *p*-value of enrichment analysis (take the negative common logarithm, i.e., −log10 *p*-value); the darker the color, the smaller the *p*-value, and the more significant the enrichment degree; the enrichment factor represents the proportion of the number of differential metabolites in this pathway in the total number of annotated metabolites in this pathway. (**B**) The small circular nodes in the metabolic pathway diagram represent metabolites; the metabolites labeled in red are the significantly upregulated differential metabolites detected in the experiment (VIP > 1, *p* < 0.05, fold change > 1), while the metabolites labeled in blue are the significantly downregulated differential metabolites detected experimentally (VIP > 1, *p* < 0.05, fold change > 1). The depth of the color indicates the degree of downward adjustment.

Since different metabolites coordinate their biological functions, the KEGG pathway-based analysis would be helpful to further understand their biological function [31]. A KEGG analysis was conducted to correlate the core metabolites identified between X and J samples (Kyoto Encyclopedia of Genes and Genomes, http://www.kegg.jp/, accessed on 30 July 2023). The KEGG pathway enrichment analysis is based on the KEGG pathway as the unit and the metabolic pathways involved in this species or closely related species as the background. Fisher’s exact test was used to analyze and calculate the significance level of metabolite enrichment in each pathway to identify the metabolic and signal transduction pathways that were significantly affected. Additionally, the KEGG enrichment pathway map between X and J samples is shown in Figure 5A. Most of the identified metabolites were mainly related to flavone and flavonol biosynthesis, flavonoid biosynthesis, isoflavonoid biosynthesis, and other pathways identified by the KEGG enrichment analysis (*p* < 0.05). The important secondary metabolites, flavones, and flavonols were also detected in all *chrysanthemum* cultivars.

The flavonoid biosynthesis pathway has been extensively investigated in different *chrysanthemum* species [32]. Flavonols belong to polyphenols, which mainly exist as glycosides in *chrysanthemum* tea, contributing to the tea’s bioactivities, bitterness, and astringency [33]. Considering the detection of flavonoids in *chrysanthemum* and previous studies on flavonoid biosynthesis pathways [34], a hypothesized *chrysanthemum* biosynthesis pathway was detected for flavonoids and flavonols (Figure 5B). As shown in Figure 5B, this pathway includes the mutual synthesis and transformation of apigenin, luteolin, two flavonoid components (glycosides), and their derivatives, along with the mutual synthesis and transformation of kaempferol, quercetin, myricetin, three flavonol components, and their derivatives. At the same time, apigenin and kaempferol can also be synthesized and transformed through the flavonoid biosynthesis pathway. To facilitate the observation of the expression of different metabolites annotated in the KEGG metabolic pathway, heatmaps of the different metabolites in the flavone and flavonol biosynthesis pathways were plotted, as shown in Figure 5C. Furthermore, quinic acid, genistein, 5-O-caffeoylshikimic acid, luteolin, apigenin, kaempferol, and naringenin were found to be the top marker metabolites for X (“HuangJu”) samples, where they were significantly more abundant than in J (“JingshihuangJu”) samples. D-malate, tryptophan, L-(-)-3-phenyllactic acid, L-malic acid, and proline were found to be the top marker metabolites for J (“JinshihuangJu”) samples, where they were significantly abundant than in X (“HuangJu”) samples.

Numerous studies have shown that flavonols and flavones are key contributors to tea infusions’ astringency and bitterness and can also significantly enhance the bitterness of caffeine [35,36]. Therefore, to statistically calculate the relationship between core metabolite compounds and taste intensity, Spearman’s correlation analysis coefficient was utilized, as shown in Figure 5D. There was a significant correlation between sensory characteristics (astringency and bitterness) and some core metabolites. According to Figure 5D, 13 core metabolites were found for the first time that could be used as quick markers for the difference in taste between the X (“HuangJu”) and J (“JinshihuangJu”) chrysanthemum teas, including 5-O-caffeoylshikimic acid, apigenin, D-glucosamine, D-malate, genistein, kaempferol, L-(-)-3-phenyllactic acid, L-malic acid, luteolin, naringenin, proline, quinic acid, and tryptophan. The main astringency contributors with tight correlations were kaempferol, luteolin, genistein, and some quinic acid derivatives. As the more common taste characteristics of *chrysanthemum* tea, these key metabolites can form various flavonol glycosides with various sugar groups to bring an astringent and convergent taste to the tea [16]. Most noteworthy, astringency is a tactile sensation caused by the interaction of astringent substances (such as polyphenols) with salivary proteins, resulting in protein precipitation and decreased lubrication in the mouth. Saliva was proven to be an oxidative agent that leads to the formation of corresponding phenolic acids [37,38]. As important bitter and astringent compounds, phenolic acid derivatives dissolve easily during oral processing, thus enhancing acidity and affecting the taste of other polyphenols. Therefore, studies on the flavonol metabolism of *chrysanthemum* tea should take into consideration that the decomposition of flavonols starts in the oral cavity. Furthermore, L-(-)-3 phenyllactic acid and L-malic acid were found to be bitter compounds in *chrysanthemum* tea. Interestingly, these compounds are also associated with the umami flavor of *chrysanthemum* tea. Umami substances have been proposed in human sensory evaluation to inhibit the bitter taste of various chemicals [39]. However, bitterness and astringency are generally undesirable; still, they are important for providing the complex sensory perceptions of *chrysanthemum* teas. Therefore, all of these are essential tasting elements of a delicious drink. According to Table 1 and Table 2 there was no significant difference in umami taste between J (“JinshihuangJu”) samples and X (“HuangJu”) samples (*p* > 0.05). In contrast, they obviously differed in bitterness and astringency (*p* < 0.05). Metabolic pathway analysis (Figure 5A,B) showed significant differences in flavonoid metabolism levels between the two varieties of *chrysanthemum* tea, which may be the reason for the difference in taste quality between the two varieties. In fact, the taste of *chrysanthemum* tea is closely related to some core chemical constituents, as shown in Figure 5D, and forms sensory qualities. Therefore, this study advances our understanding of metabolic changes, bitter taste, and astringent sensation in different varieties of *chrysanthemum* tea, and these data provide a theoretical basis for the control of sensory qualities.

## 4. Conclusions

*Chrysanthemum* tea is rich in many secondary metabolites related to its sensory qualities. This study used an untargeted metabolomics and sensory evaluation method based on UPLC-QTOF-MS and an electronic tongue to investigate key differential metabolites associated with sensory quality differences among five *chrysanthemum* (Juhua) tea varieties. A total of 1775 metabolites were identified in five varieties of *Chrysanthemum* tea by using UPLC-Q-TOF/MS analysis. The PCA, PLS-DA, and OPLS-DA results indicated significant differences in metabolome between X (“HuangJu”) samples and J (“JinshihuangJu”) samples.

To the best of our knowledge, this is the first report to reveal key taste metabolites of *chrysanthemum* tea. Of these metabolites, the contents of 13 key taste metabolites could be used for the first time as quick markers of the differences in taste between the X (“HuangJu”) and J (“JinshihuangJu”) *chrysanthemum* teas. Kaempferol, luteolin, genistein, and some quinic acid derivatives were correlated with the “astringent” attributes, while l-(-)-3 phenyllactic acid and L-malic acid were found to be responsible for the “bitterness” and “umami” in *chrysanthemum* tea. Additionally, KEGG pathway enrichment analysis showed that there were significant differences in flavonoids’ metabolism levels between X (“HuangJu”) and J (“JinshihuangJu”) *chrysanthemum* tea samples, and the pathways involved in flavonoid metabolism had important effects on the sensory qualities of different *chrysanthemum* tea varieties. Notably, this study enriches our understanding of the relationships between the key metabolites and the bitterness and astringency of *chrysanthemum* tea varieties. Untargeted metabolomics combined with electronic tongue analysis based on LC-MS can be effectively used to evaluate the differences in the sensory qualities of different varieties of *chrysanthemum* tea cultivars, which is essential for improving the quality of the finished *chrysanthemum* tea and the effective utilization of edible *chrysanthemum*. Further studies are ongoing in the authors’ lab, focusing on the formation mechanism of the key flavor components and the functional components in *chrysanthemum* tea during oral processing. We hope to report more about these advancements in the future.

## Figures and Tables

**Table 1:** Determination of taste characteristics of different varieties of *chrysanthemum* tea by electronic tongue.

	*Chrysanthemum* Varieties				
Taste Characteristics	JinshihuangJu (J)	HuangJu (X)	HanbaiJu (H)	BaoJu (B)	GongJu (G)
Sourness	−25.02 ± 0.00 ^d^	−21.36 ± 0.09 ^a^	−24.06 ± 0.09 ^c^	−21.38 ± 0.08 ^a^	−23.36 ± 0.05 ^b^
Bitterness	11.11 ± 0.00 ^b^	7.36 ± 0.01 ^e^	11.34 ± 0.01 ^a^	9.33 ± 0.01 ^d^	9.63 ± 0.02 ^c^
Astringency	13.60 ± 0.00 ^d^	15.79 ± 0.07 ^b^	14.89 ± 0.05 ^c^	16.79 ± 0.04 ^a^	14.96 ± 0.03 ^c^
Bitter Aftertaste	1.89 ± 0.00 ^a^	0.58 ± 0.03 ^e^	0.90 ± 0.05 ^c^	1.03 ± 0.05 ^b^	0.69 ± 0.02 ^d^
Astringency Aftertaste	2.79 ± 0.00 ^c^	3.36 ± 0.02 ^a^	2.03 ± 0.06 ^e^	3.00 ± 0.07 ^b^	2.60 ± 0.02 ^d^
Umami	11.88 ± 0.00 ^a^	11.79 ± 0.02 ^a^	9.55 ± 0.02 ^e^	10.25 ± 0.01 ^d^	10.30 ± 0.02 ^c^
Umami Aftertaste	2.17 ± 0.00 ^c^	3.19 ± 0.06 ^a^	1.54 ± 0.06 ^e^	2.33 ± 0.09 ^b^	1.87 ± 0.01 ^d^
Saltiness	−7.32 ± 0.00 ^b^	−2.97 ± 0.03 ^a^	−13.25 ± 0.01 ^e^	−7.74 ± 0.01 ^c^	−9.82 ± 0.06 ^d^

Standard error of means (*n* = 3). ^a–e^ Means within the same row with different superscripts differ significantly (*p* < 0.05).

**Table 2:** Traditional sensory evaluation of different varieties of *chrysanthemum* tea.

	*Chrysanthemum* Varieties				
Sensory Attributes	JinshihuangJu (J)	HuangJu (X)	HanbaiJu (H)	BaoJu (B)	GongJu (G)
Bitterness	2.90 ± 0.72 ^b^	4.13 ± 0.53 ^a^	2.70 ± 0.60 ^bc^	3.13 ± 0.67 ^b^	2.23 ± 0.65 ^c^
Astringent	2.47 ± 0.69 ^b^	3.37 ± 0.95 ^a^	2.33 ± 0.50 ^b^	2.50 ± 0.45 ^b^	2.27 ± 0.68 ^b^
Umami	1.93 ± 0.75 ^a^	1.47 ± 0.85 ^a^	2.06 ± 0.93 ^a^	1.57 ± 0.39 ^a^	2.10 ± 0.75 ^a^
Sweetness	1.60 ± 0.54 ^ab^	1.07 ± 0.14 ^c^	1.77 ± 0.57 ^a^	1.23 ± 0.42 ^bc^	1.93 ± 0.66 ^a^
Aroma	2.17 ± 0.57 ^c^	3.43 ± 0.83 ^a^	3.07 ± 0.72 ^ab^	2.23 ± 0.72 ^c^	2.43 ± 0.97 ^bc^

Each value is expressed as the mean ± SD (*n* = 10). ^a–c^ Different letters within a column indicate a significant difference (*p* < 0.05). The taste strength of each sample was evaluated using a standard scale (0, no taste; 1 to 2, slightly strong; 3 to 4, strong; 5, very strong).

**Table 3:** Contents of main bioactive substances of different varieties of *chrysanthemum* tea.

*Chrysanthemum* Varieties	Detection Index				
	Chlorogenic Acid (%)	Galuteolin (%)	Isochlorogenic Acid (%)	Total Flavonoid Content (mg GAE/g)	Total Polyphenol (mg RE/g)
JinshihuangJu (J)	1.23 ± 0.03 ^c^	1.82 ± 0.01 ^b^	2.13 ± 0.04 ^d^	116.95 ± 0.59 ^a^	53.88 ± 0.08 ^b^
HuangJu (X)	2.11 ± 0.01 ^a^	2.62 ± 0.01 ^a^	0.18 ± 0.01 ^e^	201.07 ± 0.05 ^c^	58.91 ± 0.02 ^a^
HanbaiJu (H)	1.55 ± 0.02 ^b^	0.18 ± 0.01 ^e^	4.01 ± 0.10 ^b^	106.76 ± 0.42 ^d^	38.39 ± 0.08 ^d^
BaoJu (B)	0.66 ± 0.05 ^e^	0.67 ± 0.05 ^d^	5.93 ± 0.02 ^a^	100.56 ± 0.05 ^b^	29.69 ± 0.05 ^a^
GongJu (G)	1.01 ± 0.01 ^d^	0.83 ± 0.02 ^c^	4.01 ± 0.10 ^b^	106.49 ± 0.18 ^d^	44.37 ± 0.03 ^c^

Standard error of means (*n* = 3), ^a–e^ Means within the same row with different superscripts differ significantly (*p* < 0.05).

## Data Availability

The original contributions presented in the study are included in the article/supplementary material, further inquiries can be directed to the corresponding author.

## Supplementary Materials

The following supporting information can be downloaded at: https://www.mdpi.com/article/10.3390/foods13071080/s1, Figure S1: The HPLC chromatogram of the five different varieties of *chrysanthemum* tea; Figure S2: The TIC diagram in the ESI positive and negative modes for the five different varieties of *chrysanthemum* tea; Table S1: Contents of main bioactive substances of five different varieties of *chrysanthemum* tea; Table S2: Critical Compounds Metabolites Responsible for the Metabolomics Variation Caused between X and J Samples (VIP > 1, *p* < 0.01).

## Abbreviations

Liquid Chromatography-tandem Mass Spectrometry (LC-MS/MS); Mass Spectrometry (MS); High-Performance Liquid Chromatography (HPLC); False Discovery Rate (FDR); Principal Component Analysis (PCA); Kyoto Encyclopedia of Genes and Genomes (KEGG); Traditional Chinese medicine (TCM); Gas Chromatography-Mass Spectrography (GC-MS); Headspace-gas Chromatography-ion Mobility Spectrometry (HS-GC-IMS); Gas chromatography olfactometry (GC-O); Electron Spray Ionization (ESI).
