# Supplementary material for: Distinct Changes in Metabolic Profile and Sensory Quality with Different Varieties of Chrysanthemum (Juhua) Tea Measured by LC-MS-Based Untargeted Metabolomics and Electronic Tongue
Source: Foods. 2024 Apr 1;13(7):1080. doi: 10.3390/foods13071080 (PMC11011348; doi:10.3390/foods13071080)
Supplement: Supplementary file 1 [file foods-13-01080-s001.zip › foods-2938140-supplementary.pdf]

Supplementary Material

Figure

Figure S1. The HPLC chromatogram of the five different varieties of *chrysanthemum* tea.

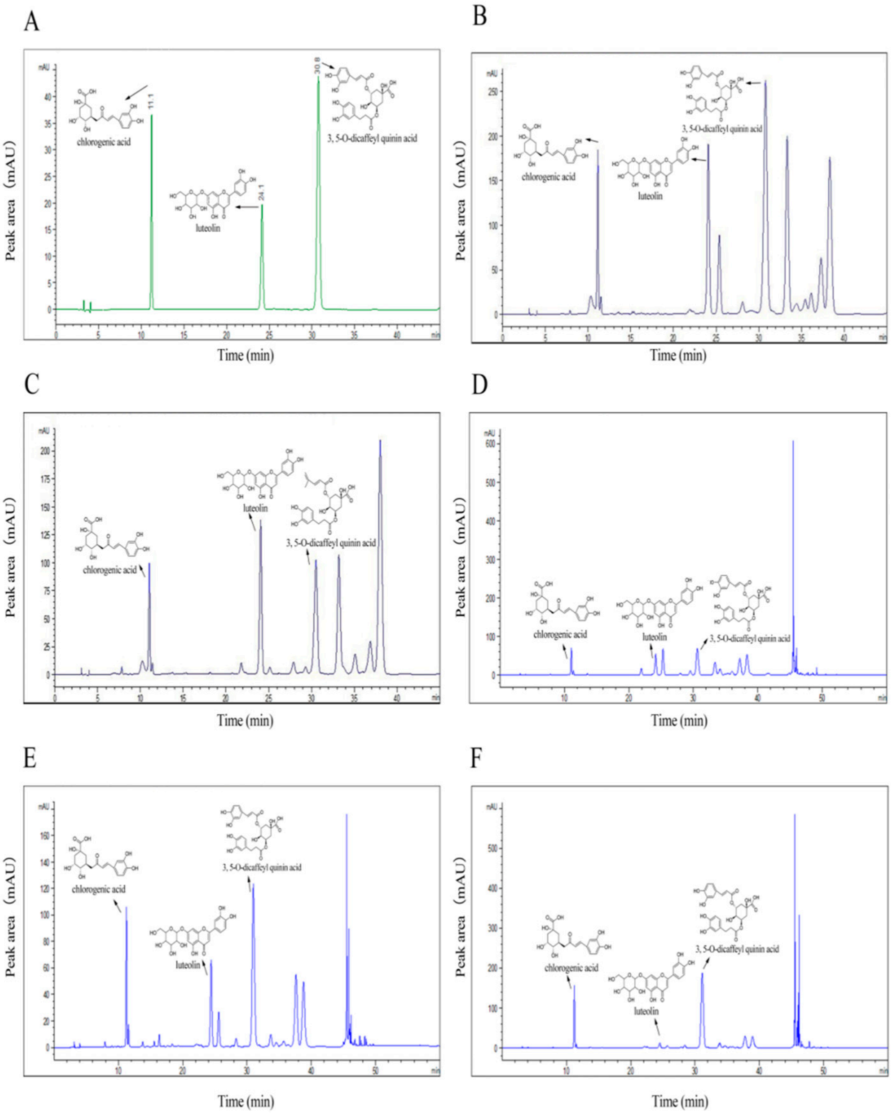

Fig.S1 The HPLC chromatogram of the five different varieties of *chrysanthemum* tea. (A) The HPLC chromatogram of the mixed sample standard (chlorogenic acid, luteolin, and3, 5-O-dicaffeyl quinin acid) ; **(B)**The HPLC chromatogram of 'Huangju' (X) O-dicaffeyl quinin acid) ; **(C)** The HPLC chromatogram of 'Jingsihuangju'(J) ;**(D)** The HPLC chromatogram of 'Boju'(B); **(E)** The HPLC chromatogram of 'Gongju'(G); **(F)** The HPLC chromatogram of 'Hangbaiju'(G);

Figure S2 The TIC diagram in the ESI positive and negative modes for the five different varieties of *chrysanthemum* tea

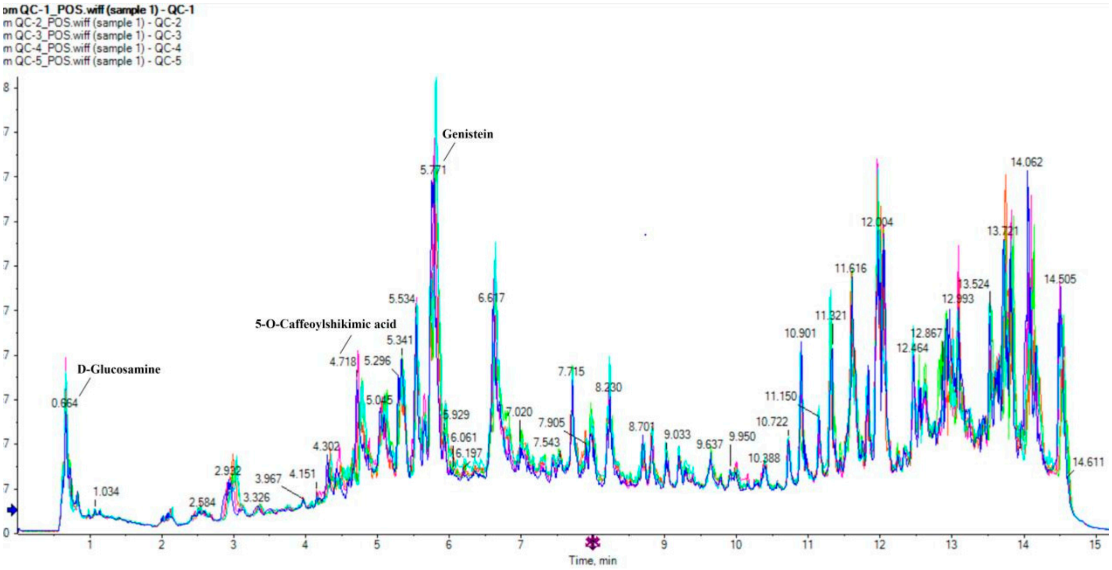

Figure S2-a The TIC diagram in the ESI positive modes for the five different varieties of *chrysanthemum* tea
(Note: The abscissa represents the retention time of each chromatographic peak, and the ordinate represents the intensity value of the peak).

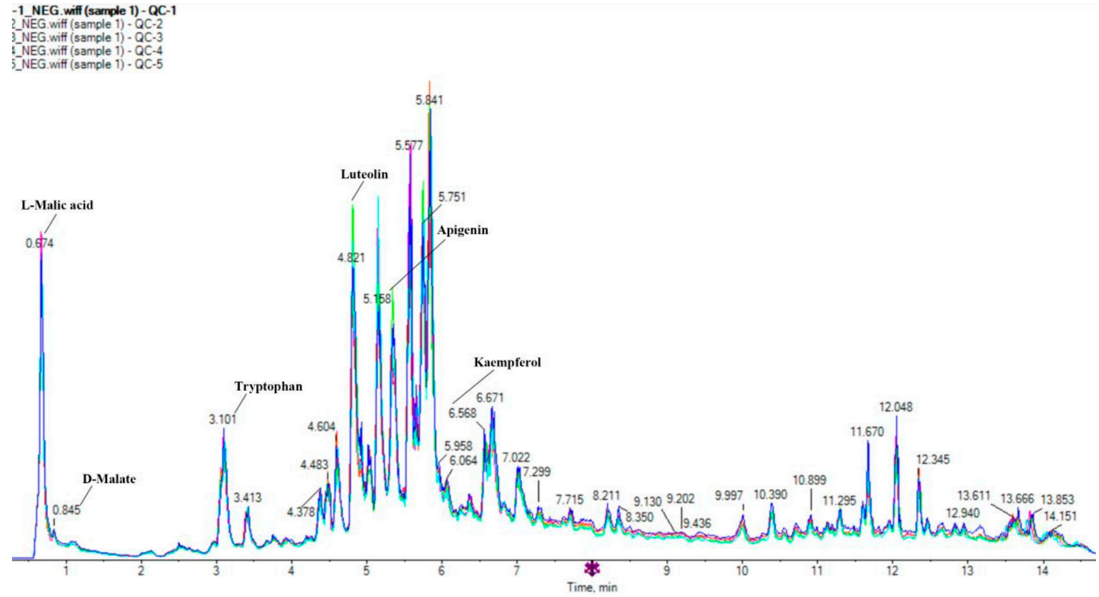

Figure S2-b The TIC diagram in the ESI negative modes for the five different varieties of *chrysanthemum* tea (Note: The abscissa represents the retention time of each chromatographic peak, and the ordinate represents the intensity value of the peak).

Table

Table S1 Contents of main bioactive substances of five different varieties of *chrysanthemum* tea

	Chrysanthemum varieties				
**Dry matter content （%）**	J	X	H	B	G
Chlorogenic acid	1.23±0.03[c]	2.11±0.01[a]	1.55±0.02[b]	0.66±0.05[e]	1.01±0.01[d]
Galuteolin	1.82±0.01[b]	2.62±0.01[a]	0.18±0.01[e]	0.67±0.05[d]	0.83±0.02[c]
Isochlorogenic acid	2.13±0.04[d]	5.93±0.02[a]	4.01±0.10[b]	1.26±0.11[e]	2.42±0.03[c]

Standard error of means (n = 3), [a-d] Means within the same row with different superscript differ significantly (P <0 .05)

Table S2 Critical Compounds Metabolites Responsible for the Metabolomics Variation Caused between X and J Samples ((VIP >1, P < 0.01)

No.	ID	Compounds	Formula	m/z	RT (min)	Superclass	VIP
1	NEG_16900	3-(Hexopyranosyloxy)-2-hydroxypropyl ester, (9Z,12Z,15Z)- 9,12,15-octadecatrienoic acid	$C_{27}H_{46}O_9$	559.30	10.976	Lipids and lipid-like molecules	1.429
2	NEG_18146	Tiliroside	$C_{30}H_{26}O_{13}$	593.12	6.595	Phenylpropanoids and polyketides	2.71
3	NEG_4360	Acacetin	$C_{16}H_{12}O_5$	283.06	6.603	Phenylpropanoids and polyketides	4.879
4	NEG_17566	Apigenin-7-O-neohesperidoside	$C_{27}H_{30}O_{14}$	577.15	5.678		3.371
5	NEG_653	Cinnamic acid	$C_9H_8O_2$	147.04	3.614	Phenylpropanoids and polyketides	1.345
6	NEG_978	L-(-)-3-Phenyllactic acid	$C_9H_{10}O_3$	165.05	3.614	Phenylpropanoids and polyketides	1.558
7	NEG_8089	Chrysosplenetin	$C_{19}H_{18}O_8$	373.09	7.691	Phenylpropanoids and polyketides	3.689
8	NEG_19601	Acacetin-7-O-rutinoside	$C_{28}H_{32}O_{14}$	637.17	6.595		1.795
9	NEG_7582	Jaceidin	$C_{18}H_{16}O_8$	359.07	7.686	Phenylpropanoids and polyketides	2.292
10	NEG_11058	(Z)-2,6-Dimethyl-7-(4-methyl-5-oxooxolan-2-yl)-3-[[3,4,5-trihydroxy-6-	$C_{21}H_{34}O_{10}$	445.20	6.76	Lipids and lipid-like molecules	1.144

		(hydroxymethyl)oxan-2-yl]oxymethyl]hept-5-enoic acid					
11	NEG_7051	5,7,3',4'-Tetrahydroxy-6,8-dimethoxyflavone	$C_{17}H_{14}O_8$	345.05	6.73	Phenylpropanoids and polyketides	1.326
12	NEG_14917	Deacetoxy(7)-7-oxokhivorinic acid	$C_{27}H_{36}O_{10}$	519.22	7.492	Organoheterocyclic compounds	2.419
13	NEG_14776	12-Acetoxyganoderic acid	$C_{30}H_{46}O_7$	517.20	7.188	Lipids and lipid-like molecules	3.522
14	NEG_11153	Astragalin	$C_{21}H_{20}O_{11}$	447.09	5.719	Phenylpropanoids and polyketides	1.868
15	NEG_6294	1-(4-Hydroxyphenyl)-3-[(2R,3R,4S,5S,6R)-3,4,5-trihydroxy-6-(hydroxymethyl)oxan-2-yl]oxypropan-1-one	$C_{15}H_{20}O_8$	327.10	3.449	Lipids and lipid-like molecules	1.293
16	NEG_467	L-Malic acid	$C_4H_6O_5$	133.01	0.667	Organic acids and derivatives	2.056
17	NEG_286	Hexanoic acid	$C_6H_{12}O_2$	115.00	0.667	Lipids and lipid-like molecules	1.195
18	NEG_4756	N-Fructosyl pyroglutamate	$C_{11}H_{17}NO_8$	290.08	0.686		1.773
19	NEG_1722	Tryptophan	$C_{11}H_{12}N_2O_2$	203.08	2.935	Organoheterocyclic compounds	1.784
20	NEG_10356	Vitexin	$C_{21}H_{20}O_{10}$	431.09	5.144	Phenylpropanoids and polyketides	1.073
21	NEG_5261	Bracteatin	$C_{15}H_{10}O_7$	301.03	6.559	Phenylpropanoids and polyketides	1.006
22	NEG_1272	2',6'-Dihydroxy-4'-methoxyacetophenone	$C_9H_{10}O_4$	181.04	6.277	Organic oxygen compounds	2.581
23	NEG_22238	PG 34:3	$C_{40}H_{73}O_{10}P$	743.48	13.79		1.185
24	NEG_280	Proline	$C_5H_9NO_2$	114.05	0.681	Organic acids and derivatives	1.898
25	NEG_11166	Maritimein	$C_{21}H_{20}O_{11}$	447.09	6.103	Phenylpropanoids and polyketides	1.221
26	NEG_4472	Luteolin	$C_{15}H_{10}O_6$	285.03	4.796	Phenylpropanoids and polyketides	1.176
27	NEG_10472	Naringenin-7-O-glucoside	$C_{21}H_{22}O_{10}$	433.11	5.482		1.334
28	NEG_10355	Apigenin-7-O-glucoside	$C_{21}H_{20}O_{10}$	431.09	5.732		9.201
29	NEG_3694	Apigenin	$C_{15}H_{10}O_5$	269.04	5.196	Phenylpropanoids and polyketides	1.199
30	NEG_4490	Kaempferol	$C_{15}H_{10}O_6$	285.03	6.563	Phenylpropanoids and polyketides	6.100
31	NEG_11024	Apigenin 7-O-glucuronide	$C_{21}H_{18}O_{11}$	445.07	5.195	Phenylpropanoids and polyketides	4.589
32	NEG_11964	Dalbinol	$C_{23}H_{22}O_8$	463.08	4.437	Phenylpropanoids and	9.344

						polyketides	
33	NEG_8114	2-[2-[(Z)-pent-2-enyl]-3-[3,4,5-trihydroxy-6-(hydroxymethyl)oxan-2-yl]oxycyclopentyl]acetic acid	$C_{18}H_{30}O_8$	373.18	6.491	Lipids and lipid-like molecules	1.100
34	NEG_3828	Naringenin	$C_{15}H_{12}O_5$	271.06	6.547	Phenylpropanoids and polyketides	1.707
35	NEG_6438	9,12,13-Trihydroxyoctadec-10-enoic acid	$C_{18}H_{34}O_5$	329.23	7.565	Lipids and lipid-like molecules	1.281
36	NEG_11149	Orientin	$C_{21}H_{20}O_{11}$	447.08	4.914	Phenylpropanoids and polyketides	1.511
37	NEG_6320	9,10-Dihydroxy-8-oxooctadec-12-enoic acid	$C_{18}H_{32}O_5$	327.21	7.342	Lipids and lipid-like molecules	1.693
38	NEG_11171	Luteolin-7-O-glucoside	$C_{21}H_{20}O_{11}$	447.09	5.353		6.826
39	NEG_466	D-Malate	$C_4H_6O_5$	133.01	0.837	Organic acids and derivatives	1.045
40	NEG_24704	Baicalin	$C_{21}H_{18}O_{11}$	891.16	5.196	Phenylpropanoids and polyketides	1.349
41	NEG_11289	Eriodictyol-7-O-glucoside	$C_{21}H_{22}O_{11}$	449.10	5.031	Phenylpropanoids and polyketides	2.142
42	NEG_3562	5,9-Dihydroxy-5,7,7-trimethyl-4,5a,6,8,8a,9-hexahydro-1H-azuleno[5,6-c]furan-3-one	$C_{15}H_{22}O_4$	265.15	6.751	Lipids and lipid-like molecules	1.459
43	NEG_19227	Quercetin 3-O-sophoroside	$C_{15}H_{10}O_7$	625.14	3.99	Flavonoid glycosides	1.387
44	NEG_4873	Glutamylphenylalanine	$C_{14}H_{18}N_2O_5$	293.12	3.246	Organic acids and derivatives	1.010
45	NEG_5743	Cirsimaritin	$C_{17}H_{14}O_6$	313.06	7.576	Phenylpropanoids and polyketides	1.205
46	NEG_11449	2-Azaniumylethyl (3-hexadecanoyloxy-2-hydroxypropyl) phosphate	$C_{21}H_{44}NO_7P$	452.27	11.601	Lipids and lipid-like molecules	1.927
47	NEG_6974	Eupatilin	$C_{18}H_{16}O_7$	343.08	7.627	Phenylpropanoids and polyketides	1.868
48	NEG_7382	Neochlorogenic acid	$C_{16}H_{18}O_9$	353.08	2.215	Organic oxygen compounds	1.943
49	NEG_4998	9-Hydroxy-10E,12Z-octadecadienoic acid	$C_{18}H_{32}O_3$	295.22	10.35	Lipids and lipid-like molecules	3.284
50	NEG_4305	trans-Vaccenic acid	$C_{18}H_{34}O_2$	281.24	12.421	Lipids and lipid-like molecules	1.740
51	NEG_7372	3-[3-(3,4-Dihydroxyphenyl)prop-2-enoyloxy]-1,4,5-trihydroxycyclohexane-1-	$C_{16}H_{18}O_9$	353.08	2.162	Organic oxygen compounds	2.032

		carboxylic acid					
52	NEG_23892	PI 34:2	$C_{43}H_{79}O_{13}P$	833.51	14.046		3.259
53	NEG_15572	Flavone base + 4O, O-MalonylHex	$C_{24}H_{22}O_{14}$	533.09	5.53	Phenylpropanoids and polyketides	5.904
54	NEG_1153	2-Isopropylmalic acid	$C_7H_{12}O_5$	175.06	2.404	Lipids and lipid-like molecules	1.234
55	NEG_1446	Quinic acid	$C_7H_{12}O_6$	191.05	0.631	Organic oxygen compounds	1.184
56	NEG_3875	FA 16:0;2O	$C_{16}H_{32}O_3$	271.22	11.353	Lipids and lipid-like molecules	1.308
57	NEG_4907	9-HOTrE	$C_{18}H_{30}O_3$	293.21	9.951	Lipids and lipid-like molecules	1.235
58	NEG_3698	Aloe-emodin	$C_{15}H_{10}O_5$	269.04	5.646	Benzenoids	5.655
59	NEG_12660	[3-[2-Aminoethoxy(hydroxy)phosphoryl]oxy-2-hydroxypropyl] octadeca-9,12-dienoate	$C_{23}H_{44}NO_7P$	476.27	11.298	Lipids and lipid-like molecules	1.001
60	POS_14378	4-Acetyl-3-hydroxy-5-methylphenyl beta-D-glucopyranoside	$C_{15}H_{20}O_8$	351.10	3.534	Organic oxygen compounds	1.370
61	POS_10508	(5xi,9xi)-12-Hydroxyabieta-7,13-dien-18-oic acid	$C_{20}H_{30}O_3$	301.21	8.211	Lipids and lipid-like molecules	1.609
62	POS_20040	Topotecan	$C_{23}H_{23}N_3O_5$	422.17	3.946	Alkaloids and derivatives	2.924
63	POS_17988	7-Hydroxy-5-methyl-2-(2-oxopropyl)-8-[3,4,5-trihydroxy-6-(hydroxymethyl)-2-oxanyl]-1-benzopyran-4-one	$C_{19}H_{22}O_9$	395.13	3.567	Organic oxygen compounds	2.593
64	POS_19227	3-O-Methylfunicone	$C_{20}H_{20}O_8$	411.10	8.237	Organic oxygen compounds	2.319
65	POS_15259	Fenofibrate	$C_{20}H_{21}ClO_4$	361.125	4.068	Benzenoids	1.086
66	POS_6344	Thunalbene	$C_{15}H_{14}O_3$	243.098	7.335	Phenylpropanoids and polyketides	1.238
67	POS_17448	Pyraclostrobin	$C_{19}H_{18}ClN_3O_4$	388.106	9.369	Organoheterocyclic compounds	3.075
68	POS_31781	Kaempferol-3,7-O-bis-alpha-L-rhamnoside	$C_{27}H_{30}O_{14}$	579.170	5.681	Phenylpropanoids and polyketides	3.348
69	POS_15557	1,17,19-Trihydroxy-7-methyl-3-oxapentacyclo[9.8.0.02,4.05,10.013,18]nonadeca-5(10),13(18),14,16-tetraene-9,12-dione	$C_{19}H_{18}O_6$	365.084	3.723	Benzenoids	1.380
70	POS_23706	Monotropitoside	$C_{19}H_{26}O_{12}$	469.131	4.675	Organic oxygen compounds	3.107
71	POS_28038	3,5,5-Trimethyl-4-[3-[(6-O-beta-	$C_{24}H_{40}O_{11}$	527.236	7.078	Lipids and lipid-like	2.002

		D-xylopyranosyl-beta-D-glucopyranosyl)oxy]butyl]-2-cyclohexen-1-one				molecules	
72	POS_21718	11,12,13-Trihydroxy-4,5,8-trimethyl-3-(2-methylpropyl)-1H,2H,3H,4H,6aH,9H,10H,11H,12H,13H,14H,15H,15bH-cycloundeca[e]isoindole-1,15-dione	$C_{24}H_{37}NO_5$	442.270	4.116	Organoheterocyclic compounds	8.167
73	POS_8718	(3S,3aR,4S,9aS,9bR)-4-Hydroxy-9-(hydroxymethyl)-3,6-dimethyl-3,3a,4,5,9a,9b-hexahydroazuleno[4,5-b]furan-2,7-dione	$C_{15}H_{18}O_5$	279.121	4.679	Organoheterocyclic compounds	1.401
74	POS_21421	Sayaendoside	$C_{19}H_{28}O_{10}$	439.151	5.727	Organic oxygen compounds	1.306
75	POS_27104	Tauromuricholic acid	$C_{26}H_{45}NO_7S$	516.306	9.139	Lipids and lipid-like molecules	1.326
76	POS_33486	(S)-7-(((2-O-6-Deoxy-alpha-L-mannopyranosyl)-beta-D-glucopyranosyl)oxy)-2,3-dihydro-5-hydroxy-2-(3-hydroxy-4-methoxyphenyl)-4H-1-benzopyran-4-one	$C_{28}H_{34}O_{15}$	611.463	12.653	Phenylpropanoids and polyketides	4.196
77	POS_21638	Simvastatin	$C_{25}H_{38}O_5$	441.257	9.904	Organoheterocyclic compounds	1.564
78	POS_18121	3',5-Dihydroxy-3,4',5',7-tetramethoxyflavone	$C_{19}H_{18}O_8$	397.086	7.894	Phenylpropanoids and polyketides	1.978
79	POS_22389	(3R,3aR,4S,6S,7aR)-6-ethenyl-4-hydroxy-3,6-dimethyl-7-[(E)-1-[(2R,3R,4S,5S,6R)-3,4,5-trihydroxy-6-(hydroxymethyl)oxan-2-yl]oxyprop-1-en-2-yl]-3,3a,4,5,7,7a-hexahydro-1-benzofuran-2-one	$C_{21}H_{32}O_9$	451.194	5.515	Lipids and lipid-like molecules	1.002
80	POS_29937	Lutein		551.414	13.622	Lipids and lipid-like molecules	1.294
81	POS_21553	3-Hydroxy-2-[[3-(3-hydroxy-6-methylheptanoyl)oxy-8-methylnonanoyl]amino]propanoic acid	$C_{21}H_{39}NO_7$	440.252	4.864	Organic acids and derivatives	15.676
82	POS_6080	[(6S,9aS)-6,9a-Dimethyl-1,3,4,5,5a,7,8,9-	$C_{15}H_{24}O_2$	237.185	8.258	Organoheterocyclic compounds	1.298

		octahydrobenzo[e][2]benzofuran-6-yl]methanol					
83	POS_6921	3a-Hydroxy-3,5a,9-trimethyl-3a,4,5,5a,6,7,8,9b-octahydronaphtho[1,2-b]furan-2(3H)-one	$C_{15}H_{22}O_3$	251.166	6.813	Lipids and lipid-like molecules	3.660
84	POS_7479	3,3a,4,5,9a,9b-Hexahydro-9a-hydroxy-6,9-dimethyl-3-methylene-, (9aR,9bS)-azuleno[4,5-b]furan-2,7-dione	$C_{15}H_{16}O_4$	261.109	4.674	Organoheterocyclic compounds	1.976
85	POS_8297	Sempervirine	$C_{19}H_{16}N_2$	273.145	8.467	Organoheterocyclic compounds	1.243
86	POS_16518	5,5,9,13-tetramethyl-7-(phenylmethylidene)tetracyclo[11.2.1.01,10.04,9]hexadec-14-en-6-one	$C_{27}H_{34}O$	375.266	11.26		1.032
87	POS_9463	(2R,3S,4S,5R,6R)-2-(hydroxymethyl)-6-(2-hydroxy-2-methylbut-3-enoxy)oxane-3,4,5-triol	$C_{11}H_{20}O_7$	287.108	2.119	Lipids and lipid-like molecules	1.676
88	POS_16713	[(3Ar,4R,5R,6E,9Z,11aR)-4-hydroxy-6,10-dimethyl-3-methylidene-2,8-dioxo-4,5,11,11a-tetrahydro-3aH-cyclodeca[b]furan-5-yl] (Z)-2-methylbut-2-enoate	$C_{20}H_{24}O_6$	378.183	7.47	Lipids and lipid-like molecules	1.158
89	POS_28471	Biochanin A 7-(6-malonylglucoside)	$C_{25}H_{24}O_{13}$	533.133	6.642	Phenylpropanoids and polyketides	4.801
90	POS_17813	Sinapaldehyde glucoside	$C_{17}H_{22}O_9$	393.111	5.05	Organic oxygen compounds	2.966
91	POS_11705	Cafestol	$C_{20}H_{28}O_3$	317.206	10.259	Organoheterocyclic compounds	1.086
92	POS_30933	Isoschaftoside	$C_{26}H_{28}O_{14}$	565.151	4.809	Phenylpropanoids and polyketides	1.237
93	POS_21669	22-Hydoxy-2-hopen-1-one	$C_{30}H_{48}O_2$	441.363	11.845	Lipids and lipid-like molecules	1.619
94	POS_23382	2-[[3,4-Dihydroxy-4-(hydroxymethyl)oxolan-2-yl]oxymethyl]-6-[(6,6-dimethyl-4-bicyclo[3.1.1]hept-3-enyl)methoxy]oxane-3,4,5-triol	$C_{21}H_{34}O_{10}$	464.246	6.769	Lipids and lipid-like molecules	1.131
95	POS_10541	2-Phenylethyl beta-D-glucopyranoside	$C_{14}H_{20}O_6$	302.159	5.092	Organic oxygen compounds	1.231
96	POS_4759	(4Ar,5R,6R)-6-hydroxy-3-	$C_{15}H_{20}O_2$	215.139	6.796	Lipids and lipid-like	1.528

		isopropenyl-4a,5-dimethyl-5,6,7,8-tetrahydronaphthalen-2-one				molecules	
97	POS_10100	9-Oxooctadeca-10,12-dienoic acid	$C_{18}H_{30}O_3$	295.224	9.473	Lipids and lipid-like molecules	1.101
98	POS_21629	N-Methyl-N-[(2E,4E,6E)-2,4,6-octatrienoyl]valylalanylprolinamide	$C_{22}H_{34}N_4O_4$	441.236	6.894	Organic acids and derivatives	1.712
99	POS_31058	4-Oxogazaniaxanthin	$C_{40}H_{54}O_2$	567.416	12.568	Lipids and lipid-like molecules	2.327
100	POS_23401	Delphinidin 3-glucoside	$[C_{21}H_{21}O_{12}]^+$	465.101	5.151	Phenylpropanoids and polyketides	1.018
101	POS_33626	5-Hydroxy-3-(4-methoxyphenyl)-7-[3,4,5-trihydroxy-6-[(3,4,5-trihydroxy-6-methyloxan-2-yl)oxymethyl]oxan-2-yl]oxychromen-4-one	$C_{28}H_{32}O_{14}$	615.165	6.606	Phenylpropanoids and polyketides	1.236
102	POS_36352	Rutarensin	$C_{31}H_{30}O_{16}$	681.1422	6.593	Phenylpropanoids and polyketides	4.461
103	POS_4919	Pymetrozine	$C_{10}H_{11}N_5O$	218.101	3.993	Benzenoids	1.033
104	POS_20872	beta-D-Glucopyranoside, 4-hydroxy-5-methyl-2-(1-methylethyl)phenyl, 6-(2-carboxyacetate)	$C_{19}H_{26}O_{10}$	432.179	5.064	Lipids and lipid-like molecules	1.376
105	POS_18049	Fucosterol	$C_{29}H_{48}O$	395.365	12.632	Lipids and lipid-like molecules	1.075
106	POS_10105	(9E,11Z)-13-Oxo-9,11-octadecadienoic acid	$C_{18}H_{30}O_3$	295.226	10.261	Lipids and lipid-like molecules	1.530
107	POS_30165	(-)-Maackiain-3-O-glucosyl-6''-O-malonate	$C_{25}H_{24}O_{13}$	555.108	6.642	Phenylpropanoids and polyketides	1.165
108	POS_15590	7,8-Dihydroxycalonectrin	$C_{19}H_{26}O_8$	365.153	5.159	Lipids and lipid-like molecules	1.372
109	POS_21418	Phenethyl 2-O-(beta-D-xylopyranosyl)-beta-D-glucopyranoside	$C_{19}H_{28}O_{10}$	439.149	5.406	Organic oxygen compounds	1.413
110	POS_22368	(2R,2S)-Eriodictyol 7-O-beta-D-glucoside	$C_{21}H_{22}O_{11}$	451.122	5.035	Phenylpropanoids and polyketides	2.060
111	POS_29451	Triferric doxorubicin	$C_{27}H_{29}NO_{11}$	544.179	5.763	Phenylpropanoids and polyketides	3.502
112	POS_4391	Sinapoyl malate	$C_{15}H_{16}O_9$	207.137	4.628	Phenylpropanoids and polyketides	1.236
113	POS_1293	Trigonelline HCl	$C_7H_7NO_2.HCl$	138.055	0.666		1.221
114	POS_32891	Violanthin	$C_{27}H_{30}O_{14}$	601.152	5.693	Phenylpropanoids and	2.430

						polyketides	
115	POS_33707	Kaempferol 3-O-beta-D-glucopyranosyl-7-O-alpha-L-rhamnopyranoside	$C_{27}H_{30}O_{15}$	617.146	5.341	Phenylpropanoids and polyketides	1.911
116	POS_29757	Flavone base + 3O, 1MeO, O-MalonylHex	$C_{25}H_{24}O_{14}$	549.116	5.943	Phenylpropanoids and polyketides	1.695
117	POS_21371	Lunarine	$C_{25}H_{31}N_3O_4$	438.231	4.844	Phenylpropanoids and polyketides	2.2391
118	POS_31471	3-{7-[5-(1-hydroxyhenicosa-4,8-dien-1-yl)oxolan-2-yl]heptyl}-5-methyl-5H-furan-2-one	$C_{37}H_{64}O_4$	573.484	13.921		2.663
119	POS_5800	Isoalantolactone	$C_{15}H_{20}O_2$	233.151	8.014	Lipids and lipid-like molecules	1.559
120	POS_5743	alpha-Pyrrolidinovalerophenone	$C_{15}H_{21}NO$	232.1679	8.268	Organic oxygen compounds	1.575
121	POS_39499	N1,N5,N10,N14-Tetra-trans-p-coumaroylspermine	$C_{46}H_{50}N_4O_8$	787.372	7.006	Phenylpropanoids and polyketides	1.5905 0
122	POS_13176	5-O-Caffeoylshikimic acid	$C_{16}H_{16}O_8$	337.084	4.758	Phenylpropanoids and polyketides	1.328
123	POS_6063	3,7-Epoxycaryophyllan-6-One	$C_{15}H_{24}O_2$	237.1826	7.572	Organoheterocyclic compounds	1.2343 67
124	POS_9312	Retinal	$C_{20}H_{28}O$	285.2187	9.92	Lipids and lipid-like molecules	1.0188 04
125	POS_6911	2-[(2R,4aS,7R)-7-Hydroxy-4a-methyl-8-methylenedecahydro-2-naphthalenyl]acrylic acid	$C_{15}H_{22}O_3$	251.1643	7.821	Lipids and lipid-like molecules	1.5261 48
126	POS_29643	3-(4-{[1,3-Dihydroxy-1-(4-hydroxy-3-methoxyphenyl)-2-propanyl]oxy}-3-methoxyphenyl)propyl 6-deoxy-alpha-L-mannopyranoside	$C_{26}H_{36}O_{11}$	547.210	4.969	Lignans, neolignans and related compounds	1.164
127	POS_29936	Echinenone	$C_{40}H_{54}O$	551.414	12.861	Lipids and lipid-like molecules	1.367
128	POS_8156	Genistein	$C_{15}H_{10}O_5$	271.059	5.753	Phenylpropanoids and polyketides	4.520
129	POS_27732	cyclo[DL-Ala-DL-Trp-DL-Ala-DL-Val-Gly]	$C_{24}H_{32}N_6O_5$	523.202	5.667	Organic acids and derivatives	1.131
130	POS_31987	Diadinochrome A	$C_{40}H_{54}O_3$	583.408	12.538	Lipids and lipid-like molecules	1.681
131	POS_27525	Anthocyanidin base+3O, O-MalonylHex	$C_{24}H_{24}O_{13}$	521.129	5.512	Phenylpropanoids and polyketides	1.595
132	POS_33324	Diosmin	$C_{28}H_{32}O_{15}$	609.180	5.784	Phenylpropanoids and polyketides	1.323
133	POS_2246	Rosmarinic acid	$C_{18}H_{16}O_8$	163.029	2.934	Phenylpropanoids and	2.958

						polyketides	
134	POS_15079	5-Hydroxy-2',4',7,8-tetramethoxyflavone	$C_{19}H_{18}O_7$	359.110	7.988	Phenylpropanoids and polyketides	2.939
135	POS_25818	Primeverin	$C_{20}H_{28}O_{13}$	499.136	3.646	Organic oxygen compounds	1.712
136	POS_27320	6''-O-Malonylgenistin	$C_{24}H_{22}O_{13}$	519.112	5.657	Phenylpropanoids and polyketides	3.044
137	POS_6214	Triacetin	$C_9H_{14}O_6$	241.065	3.398	Lipids and lipid-like molecules	1.012
138	POS_32588	Luteolin 7-O-rutinoside	$C_{27}H_{30}O_{15}$	595.165	5.321	Phenylpropanoids and polyketides	2.245
139	POS_4891	Zizanoic acid	$C_{15}H_{22}O_2$	217.158	9.424	Lipids and lipid-like molecules	1.038
140	POS_32507	Deoxykhivorin	$C_{32}H_{42}O_9$	593.277	12.467	Organoheterocyclic compounds	1.508
141	POS_3007	D-Glucosamine	$C_6H_{13}NO_5$	180.086	0.659	Organic oxygen compounds	1.007
142	POS_15169	Palatinose	$C_{12}H_{22}O_{11}$	360.150	0.677	Organic oxygen compounds	2.068
143	POS_7201	Palmitic amide	$C_{16}H_{33}NO$	256.263	11.829	Others	2.805
